# Quality Characteristics of Novel Sourdough Breads Made with Functional *Lacticaseibacillus paracasei* SP5 and Prebiotic Food Matrices

**DOI:** 10.3390/foods11203226

**Published:** 2022-10-15

**Authors:** Stavros Kazakos, Ioanna Mantzourani, Stavros Plessas

**Affiliations:** Laboratory of Food Processing, Department of Agriculture Development, Democritus University of Thrace, 68200 Orestiada, Greece

**Keywords:** sourdough bread, *Lacticaseibacillus paracasei* SP5, immobilised cells, volatiles, phytic acid, sensory evaluation

## Abstract

*Lacticaseibacillus paracasei* SP5, isolated from kefir, was assessed as a starter culture for sourdough bread making in freeze-dried form, both free (BSP5 bread) and immobilised on wheat bran (BIWB) and on a traditional flour/sour milk food, ‘trahanas’ (BITR). Physicochemical characteristics, shelf-life, volatilome, phytic acid, and sensory properties of the breads were evaluated. The BITR breads had higher acidity (9.05 ± 0.14 mL of 0.1 M NaOH/10 g) and organic acid content (g/Kg; 2.90 ± 0.05 lactic, 1.04 ± 0.02 acetic), which justifies the better resistance against mould and rope spoilage (>10 days). The highest number of volatiles (35) and at higher concentration (11.14 μg/g) were also found in BITR, which is in line with the sensory (consumer) evaluation regarding flavour. Finally, higher reduction of phytate (an antinutrient) was observed in all *L. paracasei* SP5 sourdoughs (83.3–90.7%) compared to the control samples (71.4%). The results support the use of the new strain for good quality sourdough bread.

## 1. Introduction

Among the challenges of the modern food industry, producing sourdough bread with improved sensory and nutritional properties and extended shelf-life stands out due to the growing demands of consumers for additive-free, safe, and nutritious foods. In addition, high economic loss is caused by bread wasted due to staling and microbial spoilage (mainly mould and rope spoilage) [1,2]. Innovations in bread making over the last decade include the use of enzymes [3], natural preservatives produced mainly by lactobacilli, and sourdough technology [4,5]. Indisputably, very encouraging results have been obtained by the exploitation of different types of sourdoughs, which have been used as natural bread-leavening agents since antiquity. Sourdough confers many advantages onto bread, such as improved nutritional value, health benefits, sensory characteristics, and shelf-life [5,6].

Sourdough is a complex mixture of lactic acid bacteria (LAB) and yeasts. Yeasts are responsible for the leavening activity of sourdough, while LAB contribute to ameliorated shelf-life, texture, and sensorial properties of bread through the production of various organic acids, antimicrobial metabolites, and texture conditioners [6,7,8]. Furthermore, some LAB may exhibit health-promoting properties such as antitumor, antioxidant, and anti-inflammatory properties [9]. The composition of sourdough’s microbiome markedly influences its performance in bread making. Therefore, a large number of LAB cultures have been evaluated for their capacity to confer technological improvements and distinctive characters onto sourdough bread [9]. Most of these starter cultures have been isolated from the sourdough ecosystem, but many other natural microbial consortia may also represent valuable pools for microorganisms that could be considered for sourdough production with distinctive properties, leading to high quality breads. However, the application of novel mixed or single cultures in the food industry requires process stability and standardization. Requirements for commercial application of sourdough starters, apart from conferring unique flavours and good texture, include preserved viability and functionality for long periods. Various technologies for the production of sourdough starters have been evaluated, such as freeze-drying, which is an efficient drying method for microbial cultures [10,11,12,13]. Culture immobilisation has also been established as a useful method to enhance the survival of cells during processing and storage [14]. Many studies have revealed the positive effect of immobilisation on cells’ fermentative activity, especially when the carrier is of food-grade purity and has a respectable nutritional value. In the case of LAB immobilisation in particular, carriers that are preferred are those that have prebiotic properties and contain soluble or insoluble dietary fiber enhancing the nutritional value of the produced food—such as wheat bran [11,15]. On the other hand, the selection of the immobilisation method is critical. Even though various physical methods have been proposed, the following are the those most frequently selected: (i) attachment or adsorption on solid carrier surfaces, (ii) entrapment within a porous matrix, (iii) self-aggregation by flocculation (natural), and (iv) cell containment [16]. The physical attachment or adsorption that was followed in this study is low-cost and faster than the others, with quite good results [17].

Among natural microbial sources that have been evaluated as sourdough starters, the mixed-dairy-culture kefir has been effectively applied in sourdough bread making, with interesting outcomes [18,19,20]. However, although kefir is an interesting mixed culture, the microbiota composition of kefir grains is not always stable, and standardising a process involving such a culture may be laborious—it may require daily refreshments of the sourdoughs, for example [14]. On the other hand, kefir has been evaluated as a source to isolate various LAB and yeast species with good technological and nutritional potential for various food applications, including sourdough [14,21,22,23,24,25]. For example, the recently identified strain *Lacticaseibacillus paracasei* SP5, a potential probiotic strain isolated from kefir grains [23], was used as a starter culture in white-brined-cheese production, either free or immobilized on the particles of the traditional flour and sour milk food trahanas (a starch-gluten-milk matrix) [26], with very good results regarding the quality of the products. The immobilisation matrix was used in order to protect cell viability during processing and storage and to confer a prebiotic effect onto the product. *L. paracasei* SP5 was also used successfully for chokeberry juice fermentation [27].

In this study, the strain *L. paracasei* SP5 was assessed as a starter in sourdough bread making, in freeze-dried form, free and immobilized on different matrices, to evaluate the best culture formulation that can be applied at commercial level. The produced sourdough breads were evaluated in terms of physicochemical and sensory properties, volatilome, shelf-life, and nutritional features, such as phytic acid levels.

## 2. Materials and Methods

### 2.1. Chemical Compounds Studied in This Article

Lactic acid (PubChem CID: 61503); Acetic acid (PubChem CID: 176); Butan-1-ol (PubChem CID: 263); 2-Phenylethanol (PubChem CID: 6054); Benzaldehyde (PubChem CID: 240); Ethanol (PubChem CID: 702); Ethyl acetate (PubChem CID: 8857); Furfural (PubChem CID: 7362); Isoamyl alcohol (PubChem CID: 31260); Isobutyl alcohol (PubChem CID: 6560); Formic acid (PubChem CID: 284); Propionic acid (PubChem CID: 57432895); n-Valeric acid (PubChem CID: 23616479); Caproic acid (PubChem CID: 12222599); 4-Methyl-2-pentanol (PubChem: 7910).

### 2.2. Raw Materials, Microorganism, and Media

The new, potentially probiotic strain *L. paracasei* SP5, lately isolated from kefir grains [23,28], was applied as starter culture for sourdough bread making. It was grown at 37 °C for 24–48 h in de Man–Rogosa–Sharpe (MRS) liquid broth (Fluka, Buchs, Switzerland) and the wet biomass was selected through centrifugation (Sigma 3K12, Bioblock Scientific, Illkirch-Graffenstaden, France) at 5000 rpm for 10 min at 25 °C. All media were sterilised prior to use by autoclaving at 120 °C for 15 min (1–1.5 atm).

Commercial white flour (Hellenic Biscuit CO S.A., Acharnes, Greece), and baker’s yeast (S.I. Lesaffre, Marcq-en-Barœul, France) in the form of pressed blocks (70% *w*/*w* moisture) were used in the frame of this study.

The Greek traditional, fermented food trahanas (made with wheat flour and sour milk) and wheat bran were used as immobilisation carriers of *L. paracasei* SP5 cells. Trahanas was prepared by the procedure described previously, by mixing commercial wheat flour (hard type, 70%) with sour sheep’s milk, followed by boiling, impregnation into fresh sour milk, drying/maturation for four days at 30 °C, and cutting into ~1 cm^3^ cubes [26].

Wheat bran consisted of approximately 50% dietary fibre, 20% protein, 7% ash, and 4% lipids. Initially, it was delignified by boiling with NaOH solution and sterilised by autoclaving at 120 °C, 1–1.5 atm for 15 min [29].

### 2.3. Culture Immobilisation and Freeze-Drying

For cell immobilisation by natural adsorption/attachment, 0.5 g of harvested *L. paracasei* SP5 cell mass was mixed with 5 g of trahanas or wheat bran in 500 mL MRS broth, and the mixture was incubated at 37 °C for 48 h. Subsequently, the immobilized biocatalyst was washed twice with ¼ strength Ringer’s solution for the removal of free cells. Therefore, two immobilised biocatalysts were prepared: immobilised *L. paracasei* SP5 on trahanas (ITR) and on wheat bran (IWB).

Free cells and the immobilised *L. paracasei* SP5 biocatalysts were frozen to −44 °C at a cooling rate of 5 °C/min [29] and then freeze-dried for 48 h at 5–15 mbar and at −45 °C on a FreeZone 4.5 freeze-drying apparatus (Labconco, Kansas City, MO, USA). The freeze-dried free and immobilised biocatalysts contained 8.2 ± 0.2 log cfu/g and 8.1 ± 0.3 log cfu/g of *L. paracasei* SP5 cells, respectively, and were used in the sourdough-bread-making experiments.

### 2.4. Sourdough Bread Making

For the sourdough bread making, mixing of ingredients and kneading was performed manually and the dough was moulded in 1.5 L baking pans. Initially, 3 mother sponges were prepared by mixing for 15 min 300 g wheat flour and 160 mL tap water with 1% *w*/*w* (on flour basis) of: (i) freeze-dried *L. paracasei* SP5 (free cells), (ii) freeze-dried ITR, and (iii) freeze-dried IWB. Afterwards, 3 sourdough breads were produced containing 30% *w*/*w* (on flour basis) of the aforementioned sourdoughs. The doughs of all breads contained 150 g of each sourdough, 500 g wheat flour, 270 mL tap water, and 4 g salt. In all cases, an amount of 1% *w*/*w* (on flour basis) of pressed baker’s yeast was added. All doughs were fermented at 30 °C for 2 h, proofed at 40 °C for 60 min, and baked at 230 °C for ~40 min [25]. Therefore, 3 types of sourdough breads were produced containing sourdough with: (i) freeze-dried *L. paracasei* SP5 (coded from this point on as BSP5), (ii) freeze-dried ITR (coded as BITR), and (iii) freeze-dried IWB (coded as BIWB).

Control bread was also produced with traditional sourdough (wild microflora) provided by a local bakery (coded as CB). The CB contained 30% (on flour basis) of the traditional sourdough. The recipe and procedure followed were the same as described above for the *L. paracasei* SP5 sourdoughs. All trials were carried out in triplicate.

### 2.5. Analytical Methods

#### 2.5.1. Cell Counts

The determination of the total LAB count in the bread doughs before baking was assessed as described previously [14]. Specifically, 20 g of sourdough were homogenised in 200 mL of phosphate buffer. The suspension was serially diluted, and LAB was determined on MRS agar (Fluka, Buchs, Switzerland) after incubation at 37 °C for 48–72 hours. The results are expressed as log cfu/g.

#### 2.5.2. Determination of pH and Acidity

The pH values (Sentron Argus pH-meter, Sentron Europe B.V., Roden, the Netherlands) and total titratable acidity (TTA, as volume of 0.1 M NaOH consumed/10 g sample) of the sourdough bread samples were determined as described previously [28].

#### 2.5.3. Organic Acids

Specific organic acids in the bread samples (lactic, acetic, formic, propionic, n-valeric, and caproic) were determined by ion-exchange liquid chromatography as described previously [28]. In brief, the samples were mixed with sterile water, homogenised (Seward Stomacher 400 blender, London, UK), centrifuged, and analysed on a Shimadzu HPLC system consisting of a Shim-pack ICA1 column, an LC-10AD pump, a CTO-10A oven (40 °C), a CDD-6A conductivity detector, phthalic acid (2.5 mM), and tris(hydroxymethyl)aminomethane (2.4 mM; pH 4.0) as mobile phase (1.2 mL/min). Determinations of all organic acid concentrations were carried out by means of standard curves.

#### 2.5.4. Determination of Bread Specific Volume

The loaves were weighed and the loaf volume was determined by the rapeseed displacement method [14]. The loaf volumes were calculated by deducting the rapeseed volume from the container volume. The specific volume was calculated as mL/g.

#### 2.5.5. Determination of Phytic Acid

Phytic acid (myo-inositol-1,2,3,4,5,6-hexakisphosphate) was determined enzymatically through the application of the Megazyme test-kit K-PHYT, according to the manufacturer’s recommendations (Megazyme, Bray, Ireland).

#### 2.5.6. Determination of Volatile Compounds

A semiquantitative analysis of volatile compounds (VOC) of the bread samples was also carried out. Specifically, gas chromatography/mass spectrometry (GC/MS) analysis of VOC was performed through the headspace solid-phase microextraction (SPME) sampling technique, as described before [19,28]. 4-Methyl-2-pentanol (Sigma-Aldrich, St. Louis, MI, USA) diluted in pure ethanol was used as internal standard (IS) at various concentrations (4, 40, and 400 μg/g of sample). The semiquantitative analysis of VOC was carried out by dividing the peak areas of the compounds of interest by the peak area of the IS and multiplying the result by the initial concentration of the IS. All assays were carried out in triplicate.

#### 2.5.7. Sensory Evaluation

Finally, the sensory characteristics and the recording of mould and rope spoilage in the breads were evaluated. Specifically, the sourdough breads were evaluated by 20 random, untrained testers (consumers) at a local bakery, through a blind sensory evaluation test immediately after their production, as described previously [14]. Appearance of rope and mould spoilage was evaluated by macroscopic observation [18]. The rope spoilage characteristics tested were flavour of ripe cantaloupe, discolouration, and sticky threads, while mould spoilage was determined by the characteristic appearance of visible fungi colonies.

#### 2.5.8. Statistical Analysis

Analysis of Variance (ANOVA) followed by Duncan’s post hoc multiple range test was applied to extract the specific differences between the various treatments, i.e., the effects of the different sourdoughs on the physicochemical and sensory characteristics of the produced breads. The analysis was performed using the SPSS Statistics 20.0 (IBM Corp., Armonk, NY, USA) software at an alpha level of 5%.

## 3. Results and Discussion

The application of the potential probiotic strain *L. paracasei* SP5, recently isolated from kefir [23], for sourdough bread making in freeze-dried free and immobilized form is proposed in this study. Specifically, the cells were immobilised on two food-grade supports, a starch-gluten-milk matrix (traditional food trahanas) and wheat bran, and were initially used for sourdough preparations in freeze-dried both free and immobilized forms. For comparison reasons, a traditional sourdough (control) was also used under the same preparation conditions. The physicochemical characteristics (including acidity, loaf volume, and organic acids), phytic acid content, volatilome, sensory properties, and the shelf-life of breads (regarding microbial spoilage) were evaluated, and the following results are presented and discussed.

### 3.1. Bread Volume and Acidity Levels

The results of the various physicochemical parameters of the produced breads are presented in Table 1. The specific loaf volume of all sourdough bread samples (higher than 2.4 mL/g) was similar, with no statistically significant differences, revealing that the type of starter had no effect on dough expansion, despite the presence of *L. paracasei* or the immobilisation carriers that could have affected the physicochemical properties of the dough protein network [5]. Similar bread expansion results (>2 mL/g) were obtained with another novel *Lacticaseibacillus paracasei* strain (*L. paracasei* K5, isolated from Greek cheese, and a potential probiotic), which was used in free cell form for sourdough making under the same conditions [28].

The three forms of the *L. paracasei* SP5 starter (free and immobilised on the two carriers) led to sourdough breads with statistically significant higher values of acidity (TTA) and lower pH compared to the control bread samples, which is a desired effect for candidate sourdough starters [5,28]. Specifically, the sourdoughs made with immobilised cells led to the highest TTA values (as ml 0.1 M NaOH/10 g; 8.99 ± 0.10 for BITR and 9.05 ± 0.14 g for BIWB) and the lowest pH values (4.39 for BITR and 4.38 for BIWB). According to a recent systematic review of sourdough fermentations, the average values for sourdough breads are pH 4.1 and TTA 11.0 mL of 0.1 M NaOH/10 g of dough, with the common ranges being pH 3.4–4.9 and TTA 4.0–25.0 [5]. The data obtained in this study indicate the positive role of cell immobilisation on the fermentative capacity of *L. paracasei* SP5, probably due to the retention of cell viability, catalytic activities, its protection from extremely adverse conditions of the gastrointestinal tract, and other effects on cell physiology and metabolic activity, as other researchers have reported [11,17,27]. Indeed, the total LAB count for BITR was found to be 10.5 ± 0.03 log cfu/g, while the respective values (statistically significant) for BIWB, BSP5, and CB were 9.8 ±0.02, 8.9 ± 0.02, and 8.5 ± 0.02 log cfu/g, respectively.

Especially, improvements on fermentation processes have also been previously reported regarding the immobilisation carriers used in this study (trahanas and wheat bran), which were attributed to their prebiotic properties, enhancing the viability and fermentation capacity of LAB [26,29,30,31]. Therefore, these food-grade supports seem to ameliorate the viability of *L. paracasei* SP5 boosting fermentative potential.

### 3.2. Organic Acids Content

Interesting data were recorded regarding the organic acid content of all the studied sourdough breads. In particular, BITR contained higher amounts (*p* < 0.05) of the two major organic acids (lactic and acetic acid) compared to all the other bread samples. Specifically, lactic and acetic acids were determined at concentrations of 2.90 ± 0.05 and 1.04 ± 0.02 g/Kg (or ~32.2 and ~17.3 mmol/Kg), respectively, for the BITR bread, followed by BIWB, BSP5, and CB. Sample BITR also contained higher levels of other minor organic acids (formic, propionic, valeric, and caproic). The levels of lactic and acetic acid are considered very important for the perception of sourdough flavor [32] and therefore a quotient of fermentation is usually calculated (QF, molar ratio of lactic and acetic acids) [5]. Lactic acid concentration in sourdough breads reported in the literature is usually in the range 15–150 mM, and that of acetic acid is 1–50 mM, with a QF of 0.25–20 (av. 4.4) and recommended QF below 5.0 [5]. In this study, the average QF value for all treatments was in the range 2.8–3.0.

It is also well established that lactic and acetic acids exert strong antimicrobial and antifungal effects in sourdough bread, thus they protect against mould and rope spoilage. Along with lactic and acetic acids, other acids such as formic, propionic, valeric, and caproic were determined at lower concentrations (<0.1 g/Kg). These acids have been traditionally correlated to the extended shelf-life of sourdough breads, acting synergistically with lactic and acetic acid, specifically against microbial spoilage [5,32,33,34].

### 3.3. Appearance of Mould and Rope Spoilage

**Figure 1:**
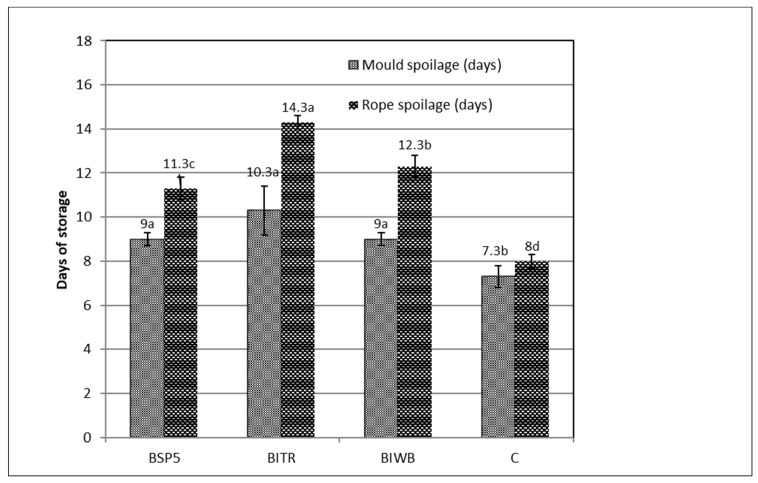
Appearance of rope and mould spoilage in the sourdough breads (BSP5: bread made with sourdough containing free, freeze-dried *L. paracasei* SP5 cells; BITR: bread made with sourdough containing ITR; BIWB: bread made with sourdough containing IWB; CB: control bread made with wild microflora sourdough. Similar lower-case letters in bars (groups for mould spoilage and groups for rope spoilage denote no significant differences at an alpha = 0.05 (ANOVA and Duncan post hoc multiple comparisons).

The appearance of mould and rope spoilage in all sourdough bread samples was assessed via daily macroscopic observations (Figure 1). The sourdough breads that contained *L. paracasei* SP5 presented better resistance to spoilage (*p* < 0.05) compared to the control sample, with BITR being the most resistant.

Specifically, rope spoilage in BITR appeared at an average of 14.3 days, followed by BIWB (12.3 days), BSP5 (11.3 days), and C (7.3 days). Mould spoilage was spotted at 10.3 days for BITR, followed by BIWB (9 days), BSP5 (9 days), and C (8 days). In general, the extended shelf-life of BITR, regarding rope and mould spoilage, could be attributed to the higher organic acids content, as discussed above and as reported by many other studies [5,28,35,36]. The higher resistance of the *L. paracasei* SP5 sourdough breads can be attributed to the high acidification level and possibly to the production of antimicrobial molecules such as bacteriocins and other metabolites that have been reported for LAB [37,38]. Specifically, the higher content of acetic acid in bread leads to higher resistance regarding mould spoilage [25,35]. Acetic acid is considered the organic acid that exhibits advanced antifungal properties. In addition, higher concentration of the minor organic acids has been reported to have positive effects regarding rope spoilage [39]. Indeed, BITR contains higher amounts of formic, propionic, n-valeric, and caproic acid (0.26 g/Kg of bread), followed by BITR and BSP5 (0.15–0.16 g/Kg of bread), and finally CB (0.04 g/Kg of bread). However, *L. paracasei* SP5 is a new strain and there are no such data available so far, making this issue an interesting subject for future research applying molecular and specified microbiological protocols.

### 3.4. Aroma Volatiles

A semiquantitative analysis of the sourdough breads for aroma-related VOC was carried out by GC-MS with a headspace SPME sampling technique. The results are presented in Table 2. Various VOC, important for sourdough flavour, were identified from three major chemical groups: alcohols, esters, and carbonyl compounds. The identified aroma VOC derive from all possible reactions that take place in the sourdough-making process, including the raw material (e.g., products of the phenylpropanoid/benzenoid pathway in plants, such as benzylalcohol and 2-phenylethanol), Maillard and Strecker degradation reactions during baking (such as furfurals, 2/3-methylbutanal, acetaldehyde, benzaldehyde, etc.), lipid oxidation and degradation products (such as alkanals), and yeast and LAB fermentation products (alcohols, esters, and carbonyls) [19,32,36,40,41,42]. The most noteworthy observation is that in all sourdough breads containing *L. paracasei* SP5, more VOC were identified at higher concentrations compared to the control sample. Specifically, the highest number (35 VOC), with a total average concentration of 11.14 μg/g, was determined in the BITR bread, followed by BSP5, and BITR (31 and 30 VOC, at 9.44 μg/g and 9.29 μg/g, respectively). In the control sample on the other hand, 22 VOC were identified, with a total average concentration of 7.65 μg/g (Table 2). Among the VOC determined, a significant group of compounds were comprised of esters, which are generally considered to have a positive effect on food aroma with their fruity, flower, and honey notes.

As can be seen from Table 2, BITR contained the identified esters at higher total (average) concentration (1.2 μg/g), followed by BIWB (1.08 μg/g) and BSP5 (0.93 μg/g), and with much lower amounts found in the control bread (0.27 μg/g). The most statistically significant differences were also observed in the case of individual compounds, which were generally found at lower levels in the control bread (*p* < 0.05), except for ethanol, benzylalcohol, 2-phenylethanol, hexanal, and furfural, which were found at similar levels (*p* < 0.05) in all samples. Ethanol is the main product of alcoholic fermentation and may be a product of LAB heterofermentative metabolism [32]. Benzylalcohol and 2-phenylethanol, as well as 3-methylbutanal (found mainly in BITR and BSP5), are among the compounds that have been related to intense bready aromas [32]. These observations indicate the positive role of *L. paracasei* SP5 in the complexity of bread aroma, although the number of VOC in a food matrix does not necessarily confer a positive aroma impact [43]. According to Arora et al., the available literature on sourdough research clearly correlates VOC prevalence with the dominant LAB and yeast species in sourdoughs, indicating a species- specific synthesis of VOC [5]. However, the addition of yeasts (*Saccharomyces* or other) in sourdoughs is known to strongly affect the production of alcohols, esters, and specific carbonyl compounds, therefore the volatile profile of sourdough breads will also be strongly determined by the type and amount of added yeast, the processing conditions that affect yeast growth and metabolism, as well as the type of raw material and the baking process that leads to VOC losses and chemical transformations [32].

### 3.5. Phytic Acid Content

**Figure 2:**
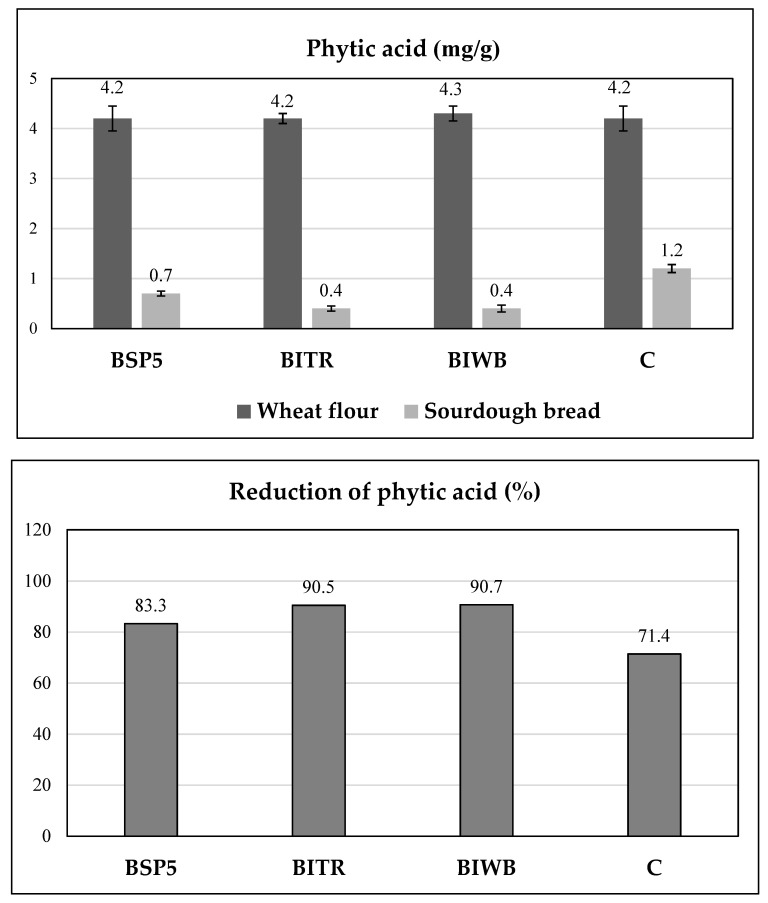
Phytic acid content (up) and phytic acid reduction (down) in doughs before and after baking (BSP5: bread made with sourdough containing free, freeze-dried *L. paracasei* SP5 cells; BITR: bread made with sourdough containing ITR; BIWB: bread made with sourdough containing IWB; CB: control bread made with wild microflora sourdough).

Phytic acid is a known antinutrient in bread that is related to reduced mineral bioavailability [41]. The phytic acid levels and degradation ratios in the sourdough breads made in this study are illustrated in Figure 2. Although it is well established that sourdough fermentation is more effective than yeast fermentation in decreasing the phytate content in bread [44], significant differences were observed between all the produced sourdough breads in this study. The highest phytic acid level (1.2 mg/g) was determined in the control bread (CB), while all sourdough breads that contained free or immobilised *L. paracasei* SP5 cells (BSP5, BITR, and BIWB) presented lower values (0.4–0.7 mg/g). Likewise, the reduction of phytic acid was higher in the BSP5, BITR, and BIWB samples (av. 83.3–90.7%), with the respective value for the CB sample being 71.4%. Specifically, the sourdough breads that contained immobilised *L. paracasei* SP5 (BITR and BIWB) presented the highest reduction of phytic acid (*p* < 0.05) compared to BSP5 and CB (Figure 2).

This could be explained by the fact that either *L. paracasei* SP5 exhibits significant extracellular phytase activity, as other researchers have proposed for LAB, or the reduction of phytate is due to activation of the intrinsic cereal phytases by the decreased pH of the sourdough breads (Table 1) [44]. In conclusion, the use of *L. paracasei* SP5 seems to increase the nutritional value of bread, based on the significant reduction of phytic acid.

### 3.6. Sensory Evaluation (Consumer Preference)

The results of the preliminary sensory evaluation (consumer preference) of the produced sourdough breads are presented in Table 3. No statistically significant differences were observed regarding the sensory parameters evaluated among the different samples, except for the score on flavour. Specifically, BITR received a better score for flavour (*p* < 0.05) compared to all other samples, which is commensurate with the findings of the GC-MS analysis of VOC. In conclusion, the three bread samples that contained *L. paracasei* SP5 (free or immobilized) seem to be accepted similarly to the commercial sourdough bread by the consumers.

## 4. Conclusions

The new, potentially probiotic strain *L. paracasei* SP5, recently isolated from kefir, was effectively used as a starter culture in sourdough bread making. Moreover, cell immobilisation on wheat bran and trahanas (acting as protective, prebiotic matrices) positively influenced the fermentation capacity of the bacteria. Especially the sourdough breads made with cells immobilised on trahanas (BITR) presented higher acidity levels and organic acid content, which justifies their better resistance against mould and rope spoilage. Higher number of volatiles at higher total concentration were also identified in BITR, which is in line with the sensory (consumer) evaluation regarding flavour. Finally, higher reduction of phytate (an antinutrient) was observed in all the *L. paracasei* SP5 sourdoughs compared to the control bread, supporting the use of the new strain for good quality sourdough bread.

## Figures and Tables

**Table 1:** Physicochemical characteristics of breads made with sourdoughs prepared with freeze-dried free and immobilized *L. paracasei* SP5 and with the control sourdough.

Bread Sample	pH	TTA /*-(mL 0.1 M NaOH/10 g)	SLV /*-(mL/g)	Organic Acids (g/Kg Bread)					
				Lactic	Acetic	Formic	Propionic	n-Valeric	Caproic
BSP5	4.60 ± 0.04 ^b^	7.49 ± 0.07 ^b^	2.49 ± 0.07 ^a^	2.51 ± 0.07 ^b^	0.89 ± 0.01 ^c^	0.04 ± 0.01 ^b^	0.04 ± 0.01 ^a^	0.04 ± 0.01 ^b^	0.04 ± 0.01 ^a^
BITR	4.39 ± 0.05 ^a^	8.99 ± 0.10 ^a^	2.54 ± 0.05 ^a^	2.90 ± 0.05 ^a^	1.04 ± 0.02 ^a^	0.08 ± 0.01 ^a^	0.05 ± 0.01 ^a^	0.08 ± 0.01 ^a^	0.05 ± 0.01 ^a^
BIWB	4.38 ± 0.05 ^a^	9.05 ± 0.14 ^a^	2.54 ± 0.06 ^a^	2.75 ± 0.05 ^b^	0.96 ± 0.03 ^b^	0.05 ± 0.01 ^b^	0.04 ± 0.01 ^a^	0.03 ± 0.01 ^b^	0.03 ± 0.01 ^b^
CB	4.73 ± 0.05 ^c^	6.73 ± 0.08 ^c^	2.58 ± 0.04 ^a^	2.28 ± 0.07 ^c^	0.77 ± 0.02 ^d^	0.04 ± 0.01 ^b^	tr	tr	tr

BSP5: bread made with sourdough containing free, freeze-dried *L. paracasei* SP5 cells; BITR: bread made with sourdough containing ITR; BIWB: bread made with sourdough containing IWB; CB: control bread made with wild microflora sourdough; TTA: total titratable acidity; SLV: specific loaf volume; tr: traces (<0.01 g/Kg); Different superscript letters in a column indicate statistically significant differences (ANOVA, Duncan’s multiple range test, *p* < 0.05).

**Table 2:** SPME GC/MS analysis of aroma-related compounds (μg/g) of breads made with sourdoughs containing freeze-dried free and immobilized *L. paracasei* SP5 and with the control sourdough.

KI	Compound	RI	Concentration (μg/g)			
			BSP5	BITR	BIWB	CB
Alcohols						
832	Ethanol	A	4.09 ± 0.23 ^a^	4.11 ± 0.12 ^a^	3.95 ± 0.11 ^a^	4.23 ± 0.18 ^a^
1012	Isobutyl alcohol	A	0.10 ± 0.01 ^b^	0.19 ± 0.02 ^a^	tr	0.10 ± 0.03 ^b^
1120	Isoamyl alcohol	A	0.19 ± 0.05 ^b^	0.29 ± 0.06 ^a^	0.30 ± 0.07 ^b^	0.14 ± 0.05 ^b^
1160	Butan-1-ol	A	0.14 ± 0.02 ^b^	0.25 ± 0.02 ^a^	0.21 ± 0.02 ^a^	0.11 ± 0.03 ^b^
1230	Pentan-1-ol	B	0.21 ± 0.02 ^a^	0.27 ± 0.05 ^a^	0.21 ± 0.04 ^a^	0.14 ± 0.02 ^b^
1257	Hexan-1-ol	A	0.13 ± 0.02 ^b^	0.11 ± 0.01 ^b^	0.10 ± 0.02 ^a^	0.13 ± 0.01 ^a^
1435	Heptan-1-ol	B	0.07 ± 0.01 ^b^	0.16 ± 0.03 ^a^	0.10 ± 0.02 ^b^	0.05 ± 0.02 ^b^
1466	Octan-1-ol	A	0.17 ± 0.02 ^b^	0.23 ± 0.02 ^a^	0.24 ± 0.03 ^a^	nd
1480	Heptan-2-ol	A	0.07 ± 0.01 ^a^	0.08 ± 0.01 ^a^	0.10 ± 0.02 ^a^	nd
1540	1-Octen-3-ol	B	0.10 ± 0.02 ^a^	0.16 ± 0.02 ^a^	0.14 ± 0.03 ^a^	nd
1670	Benzylalcohol	A	0.18 ± 0.03 ^a^	0.19 ± 0.02 ^a^	0.17 ± 0.04 ^a^	0.18 ± 0.03 ^a^
1812	2-Phenylethanol	A	0.29 ± 0.03 ^a^	0.37 ± 0.05 ^a^	0.31 ± 0.06 ^a^	0.27 ± 0.06 ^a^
Esters						
<800	Ethyl acetate	A	0.21 ± 0.05 ^b^	0.37 ± 0.04 ^a^	0.19 ± 0.02 ^b^	0.18 ± 0.03 ^b^
1107	Butyl acetate	A	0.5 ± 0.01 ^b^	0.11 ± 0.02 ^a^	tr	0.05 ± 0.01 ^b^
1162	Hexyl acetate	B	tr	0.08 ± 0.02	tr	nd
1250	Ethyl pentanoate	B	tr	0.05 ± 0.01 ^a^	0.03 ± 0.01 ^a^	nd
1395	Ethyl hexanoate	B	nd	0.07 ± 0.01 ^a^	0.05 ± 0.01 ^a^	nd
1438	Ethyl octanoate	B	nd	0.07 ± 0.01 ^a^	0.04 ± 0.01 ^b^	nd
1445	Ethyl heptanoate	A	nd	0.08 ± 0.01 ^a^	0.05 ± 0.01 ^b^	nd
1590	Isobutyl acetate	B	0.04 ± 0.01 ^c^	0.10 ± 0.01 ^a^	0.07 ± 0.01 ^b^	0.04 ± 0.01 ^c^
1848	Ethyl dodecanoate	B	0.05 ± 0.01 ^b^	0.08 ± 0.01 ^a^	0.05 ± 0.01 ^b^	nd
1850	2-Phenylethyl acetate	B	0.04 ± 0.01 ^b^	0.08 ± 0.01 ^a^	0.05 ± 0.01 ^b^	nd
2410	Ethyl octadecanoate	B	0.05 ± 0.01 ^a^	0.06 ± 0.01 ^a^	0.5 ± 0.01 ^a^	tr
2429	Ethyl 9-octadecenoate	B	0.04 ± 0.01 ^a^	0.05 ± 0.01 ^a^	0.05 ± 0.01 ^a^	nd
Carbonyl compounds						
<800	Acetaldehyde	B	0.18 ± 0.04 ^b^	0.29 ± 0.03 ^a^	0.19 ± 0.03 ^b^	0.10 ± 0.01 ^c^
812	2-Methylbutanal	B	0.05 ± 0.01 ^b^	0.10 ± 0.01 ^a^	0.11 ± 0.01 ^a^	0.05 ± 0.01 ^b^
986	3-Methylbutanal	A	0.15 ± 0.03 ^a^	0.19 ± 0.02 ^a^	0.10 ± 0.01 ^b^	0.09 ± 0.01 ^b^
1002	Hexanal	A	0.14 ± 0.02 ^a^	0.12 ± 0.01 ^a^	0.15 ± 0.03 ^a^	0.13 ± 0.01 ^a^
1080	Heptanal	A	0.09 ± 0.02 ^a^	tr	0.11 ± 0.02 ^a^	tr
1100	2,3-Butanedione (diacetyl)	B	0.08 ± 0.01 ^a^	0.09 ± 0.02 ^a^	nd	nd
1334	Furfural	A	0.21 ± 0.02 ^a^	0.24 ± 0.02 ^a^	0.19 ± 0.02 ^a^	0.22 ± 0.05 ^a^
1358	Nonanal	B	0.09 ± 0.01 ^b^	0.15 ± 0.02 ^a^	0.08 ± 0.01 ^b^	0.04 ± 0.02 ^c^
1448	γ-Butyrolactone	B	1.17 ± 0.07 ^c^	1.76 ± 0.09 ^a^	1.24 ± 0.05 ^b^	1.09 ± 0.08 ^c^
1458	Benzaldehyde	A	0.34 ± 0.04 ^a^	0.36 ± 0.05 ^a^	0.21 ± 0.04 ^b^	0.19 ± 0.05 ^b^
1541	2-Nonenal	B	0.09 ± 0.01 ^a^	0.09 ± 0.01 ^a^	tr	0.04 ± 0.01 ^b^
1582	5-Methylfurfural	B	0.18 ± 0.03 ^a^	0.14 ± 0.03 ^a^	tr	0.08 ± 0.02 ^b^

BSP5: bread made with sourdough containing free, freeze-dried *L. paracasei* SP5 cells; BITR: bread made with sourdough containing ITR; BIWB: bread made with sourdough containing IWB; CB: control bread made with wild microflora sourdough; KI: Kovats Index; RI: Reliability of Identification; A: positive identification by MS data and retention times and those of standard compounds; B: positive identification by MS data only; tr: traces (<0.01 g/Kg); nd: not detected; Different superscript letters in a row indicate statistically significant differences (ANOVA, Duncan’s multiple range test, *p* < 0.05).

**Table 3:** Sensory evaluation test (consumer preference) of breads made with sourdoughs prepared with freeze-dried *L. paracasei* SP5 and with the control sourdough.

Sourdough Bread Sample	Flavour	Taste	Appearance	Overall Quality
BSP5	8.6 ± 0.1 ^b^	8.5 ± 0.2 ^a^	8.3 ± 0.1 ^a^	8.6 ± 0.2 ^a^
BITR	8.9 ± 0.1 ^a^	8.6 ± 0.1 ^a^	8.3 ± 0.2 ^a^	8.7 ± 0.1 ^a^
BIWB	8.6 ± 0.1 ^b^	8.5 ± 0.2 ^a^	8.4 ± 0.1 ^a^	8.6 ± 0.1 ^a^
CB	8.5 ± 0.1 ^b^	8.5 ± 0.1 ^a^	8.4 ± 0.1 ^a^	8.7 ± 0.1 ^a^

BSP5: bread made with sourdough containing free, freeze-dried *L. paracasei* SP5 cells; BITR: bread made with sourdough containing ITR; BIWB: bread made with sourdough containing IWB; CB: control bread sample made with wild microflora sourdough; Different superscript letters in a column indicate statistically significant differences (ANOVA, Duncan’s multiple range test, *p* < 0.05).

## Data Availability

Data is contained within the article.
